# M. Louis' Curious Case of Sub-Acute Metritis Combined with Phlebitis

**Published:** 1826-07

**Authors:** 


### SUBACUTE METRITIS, WITH INFLAMMATION OF THE VEINS.—M. LOUIS.

Nothing is more easy, observes M. Louis, than to distinguish inflammation of the uterus, if we believe the symptomatology of systematic authors. They will even inform you how to recognize the particular portion of the organ that is phlogosed. But we may search in vain for the facts on which their symptomatology is founded. It would, therefore, be very desirable, that those who have the charge of public institutions for females should communicate to the public the details of such cases as come under their observation, in order that the phenomena of female complaints might be more clearly connected with the pathological conditions on which they depend. We shall now proceed to the curious case under consideration.

An unmarried woman, 27 years of age, of apparently strong constitution, was admitted into La Charité, on the 3d January, 1826, having been safely delivered of a child twenty days previously. The day after delivery she was seized with head-ache, cold chills, pains in the hypogastrium, and slight diarrhœa. These symptoms persisted, the cold chills coming on every day. There was no nausea for the first fortnight; but the thirst was urgent, anorexia complete, the lochia moderate. The urine was very red, and passed with difficulty. Nothing had been prescribed but diluent drinks and lavements. *4th January.* Her countenance was expressive of malaise and even of pain—complained of great debility—pain in the right thigh through its whole extent, for four days past, exasperated by pressure and motion—tongue dry and hard—mouth pasty—thirst intense—anorexia and nausea—abdomen soft, except in the right side, low down, where a roundish tumour could be felt, about three inches in diameter, little painful unless pressed. No pain on pressure in any other part of the abdomen—slight uterine discharge—pulse regular and 120 in the minute—animal temperature moderate. *Thirty leeches to the hypogastrium—fomentations—starvation.* Nothing particular on the succeeding day. *6th.* The restlessness and debility had increased—the eyes and the whole skin had become yellow—speech difficult—large hæmorrhoidal tumour at the anus—the volume of the uterus found to be larger than natural—the os tincæ soft, and very little painful to the touch—tumour in the right hypogastrium did not seem connected with the womb, as no motion was communicated to it, when the uterus was elevated. *7th,* The intensity of the yellow colour was increased—the countenance depressed—the hypogastrium very painful, as was the abdomen generally. *8th,* All the symptoms were ameliorated; but the abdomen seemed larger in size. This amendment was of very short duration, and the patient was in a state of stupor most part of the day. In this condition she lingered till the *12th*, when she expired.

*Dissection.* We shall pass over the examination of the head and chest, as not offering any thing worthy of notice, and come at once to the abdomen. There was nothing remarkable at first sight in this region. But on moving the intestines, a large quantity of pus was found in the pelvis, of a yellow colour and homogeneous appearance. All the parts with which the pus was in contact were covered with false membrane, beneath which the peritoneum was seen red and livid. The intestines in the pelvis were glued together. The uterus was larger than natural by one half, and, both internally and externally, of a rosy red-colour. The parenchymatous structure of the organ was softened—and whereever it was cut with the scalpel there were seen canals which threw out a quantity of pus of a very yellow colour. On tracing these canals, by means of a probe, they were found to lead to a double tumour beyond the uterus, one of which was an inch and a half in diameter. These tumours consisted of a conglomeration of canals, such as have been described, and filled with pus. From this mass was traced one large canal, nine inches in length, which opened into the cava inferior, below the emulgent veins. This canal, which was no other than the common trunk of the uterine and ovarian veins of the right side, was ten lines in circumference in the greater part of its length, and only half that at its entrance into the cava. It contained pus throughout its whole extent, and was lined with a fine false membrane. The uterine and ovarian veins were much dilated, and thickened in their parietes. No pus could be found in the cava itself. The mucous membrane of the bladder was in a state of inflammation, and covered with a false membrane of a very yellow colour.

We shall not follow M. Louis in his reflexions on this curious case, but leave it to the reflexions of our readers. The public situation in which the case happened, and the character of the reporter cannot admit of the least doubt of the authenticity of the facts above stated, which are certainly of a very interesting nature in a pathological point of view.—*Archives, Mars.* 1826.

